# 

**Published:** 1898-05-14

**Authors:** 


					THE HOSPITAL. "The standard of highest purity."?Lancet. May 14th, 1898.
CADBURY'S m&k Wjr tes* CADBURY'S
cocoa WnlL cocoa
ABSOLUTELY PURE. r*1? i NO DRUGS OR ALKALIES USED.
No. 6g7. Yol. XXIV. Saturday,
May 14th, 1898.
[SuhBcription^TonttH,"! pRICB TWOPENCE.

				

